# The Rational Treatment of Asthenopia1Read at the annual meeting of the Medical Association of Central New York, at Rochester, October 20, 1896.

**Published:** 1897-03

**Authors:** Wheelock Rider

**Affiliations:** Rochester, N.Y. 53 South Fitzhugh Street


### THE RATIONAL TREATMENT OF ASTHENOPIA.[1]

By WHEELOCK RIDER, M. D., Rochester, N. Y.

AFTER a period of labor, its length depending upon the local and general muscular development, the general condition of the individual and the amount of power expended, there arises in all voluntary muscles an indefinable sensation of exhaustion, described as "tiredness," which induces rest from effort. Now, it is a physiological fact that in doing close work the eyes are entirely dependent upon muscular effort, and in so far are subject to the same laws of endurance and fatigue as are other muscle groups.

The term asthenopia comprises several symptoms of muscular fatigue—namely, localised pain, dimness of vision in so far as it depends upon accommodation, congestion of the conjunctiva, drowsiness, intolerance of light, and several other reflexes. Asthenopia, then, is to be considered as pathological in that a normal discomfort is experienced after less effort than suffices to fatigue a normal muscle; in other words, asthenopia is a relative incapacity for ocular muscular endurance.

Time will not permit me to trace, so fully as I would wish, the growth of our acquaintance with the motor apparatus of the eye. Briefly, the knowledge of the function of accommodation dates from the middle of this century. For thirty years refractive errors, pure and simple, received attention from the surgeon, while during the last fifteen years much study has been given by some observers to the vagaries of the extrinsic muscles.

I wish to call your attention to a method of treatment which is now much in vogue for the relief of asthenopia—namely, by the so-called graduated tenotomy of the extrinsic muscles. I hope to show by a history, typical of a very great number—an honest average case—that such treatment is physiologically irrational, that the attempts are often futile and the results sometimes disastrous. I will present the detailed history of this case, and in a brief discussion of it, will elucidate the views on the treatment of asthenopia which alone appear to me rational.

In June, 1895, a young woman consulted me, bearing an introductory letter from a member of her family, telling me of her having been under the care, for nearly five years, of an ophthalmic surgeon in another part of the state. The complete notes of her

1. Read at the annual meeting of the Medical Association of Central New York, at Rochester, October 20, 1896.

case, taken by her previous consultant, were enclosed. Her medical adviser I know to be carefully prepared and fully equipped for his work. He is a man of large practice and much experience, and in his view of the case he may be considered to fairly represent, I am sorry to say, a too large number of the ophthalmic surgeons of today. Following is a copy of his notes of her case :

Miss X., aged 20 years ; first seen November 12, 1890. Symptoms, headache between the eyes, asthenopia, vertigo, nausea and some mental symptoms. Vision, right eye, $\frac{6}{8}$ ; with + .50 sph. and + .25 cyl. 90° vision is $\frac{6}{5}$. Vision, left eye, $\frac{6}{6}$ ; with + 1. sph. and + .25 cyl. 90° vision is $\frac{6}{5}$. Latent divergence, 9°. Abduction, 15°. Adduction, 20°. With red glass over one eye, momentary diplopia. Homatropine gave same refraction, which was corrected, with 2° prism base in, added. Worn a month with seeming benefit, but prisms being inadequate, tenotomy was advised.

December 12, 1890. Partial tenotomy of left external rectus, exophoria being previously 9°.

January 24, 1891. Exophoria still 7° or 8°. but about all latent. Abduction still 15°. Partial tenotomy of right external rectus, leaving esophoria of less than 1°.

February 21, 1891. Exophoria, 4°, and a partial tenotomy of left was done.

April 10, 1891. Much relieved. For two weeks after last operation was free from headache. No latent deviation. Left hyperphoria, ½°, this being the first indication of hyperphoria.

October 13, 1891. Orthophoria.

March 29, 1892. Some headache and dizziness ; cannot use eyes much, but during last winter much improved in general health. Exophoria, 5° by phorometer, but there is probably much more, as the divergent movement seen in covering one eye is marked. Exophoria corrected by tenotomies, each being followed by a period of relief, the last leaving 2° or 3° of esophoria.

February 28, 1893. Esophoria, ½°. Glasses now changed to + 1. sph., with + .50 cyl. 90° each eye.

July, 1893. Cannot use eyes. Exophoria 2° or 3°. Prisms added to the glasses, without relief.

October 6, 1893. Left hyperphoria, ¾°, and she accepts : right, + 1.25 sph. with + .50 cyl. 90° ; left, + 1.50 sph. with + .50 cyl. 90°. Since October refraction has not changed. These glasses ordered, decentered to correct hyperphoria.

February, 1894. Headaches returned.

March, 1894. Left hyperphoria, 2½° to 3°. Exophoria, 1½°. Abduction, 8°. Corrected by decentration, 2½°.

May 9, 1894. Left hyperphoria, 4°. Prisms ordered.

June 13, 1894. Headaches not relieved. Left hyperphoria, 4°. Partial tenotomy of left superior rectus, leaving 1° ; later, no hyperphoria.

December 7, 1894. Still complains of headache. Exophoria, 4° ; no hyperphoria. Gave prisms to exercise internal recti, but without benefit.

January, 1895. Exophoria, 2° or 3° ; no hyperphoria.

This narrative of so much careful labor on the part of the surgeon and of such long suffering and endurance on the part of the patient seems to need no further comment. I would ask you to note that after more than three years and numerous operations the deviation remains the same ; that, notwithstanding an almost diurnal variation which is known to exist, we have, in December, 1894, an exophoria of 4°, as in February, 1891. In March, 1892, we had, after several (number not stated) tenotomies, an esophoria of 3°, gradually changing in sixteen months, without operation, into an exophoria of 3°.

The patient told me that she had made no effort to use her eyes for close work during the past two years, and she complained bitterly of her enforced idleness. The glasses with which she was provided I found to be correct. I did not investigate the unbalanced extrinsic (recti and oblique) muscles. In a general conversation, during which I could closely observe her temperament and disposition, I learned that she was, in a sense, a spoiled child, rather neurotic, overworked by society's demands, very despondent as to getting relief, but altogether intelligent and likely to follow advice. She was shown models and plates of the eye, and was interested in some simple experiments, all demonstrating the motor physiology of vision, after which she returned home instructed to read one minute in the forenoon at a given hour, and again one minute in the afternoon at a given hour—another person to act as timekeeper. On the second day the limit was set at two minutes ; on the third day at three, and so on until a half-hour's reading was accomplished, when she was to rest fifteen minutes and proceed as before. She was to wear her correcting glasses for close work only. She was to have several hours' employment out of doors each day. Her recovery was uneventful, and she was discharged reading daily three hours systematically, and as much more as pleased her. The management of this case by her former adviser is not unusual in the following items : he failed in the beginning to discover the full amount of her hypermetropia and astigmatism. He believed that a lack of balance of the strong recti muscles explained her asthenopia, and he thought he could operatively weaken a strong muscle more accurately than the opposing muscle could strengthen itself by the labor necessarily put upon it if single vision was to be maintained. He failed to apply the doctrine to which he must assent—that function, in a general way, determines the organ, that the blacksmith's hammer is the father of his biceps.

My view of the case was, that no matter how far, within reasonable limits, he had, by so-called graduated tenotomies, upset the balance of the extrinsic directing muscles, the necessity for avoiding diplopia would compel her muscles to preserve the proper coördination, and that a compensatory development would follow. I held, moreover, that the ciliary muscle was weak from disuse when the patient came to me, that by exercise it would develop strength —remembering that in myopic eyes in which accommodation is not required, there are often no circular fibers whatever, and that in hypermetropic eyes the circular fibers are developed to constitute, in some instances, half the bulk of the entire muscle. I believed, moreover, that with well-trained adjusting apparatus she would be enabled to endure the labor which her oversight put upon her for distance, and that she might lay aside her glasses, but that in training it was well to place her eyes upon a physiological basis and obliterate, for a time, her deformity.

We who are not myopic have, as a rule, hypermetropia ; we are very generally astigmatic, and eighty-four of us in every hundred have unbalanced recti muscles. On the other hand, relatively few of us suffer from asthenopia. It is not unusual to find individuals whose eyes, with hypermetropia of two dioptries, have never for thirty-five years given the least evidence of the defect, and very recently there has come under my observation an instance of marked asymmetrical development of the recti muscles which nicely illustrates how great a tolerance in this regard may be established.

A young woman whose history was clear of all suspicion of eye-strain had an embolism of the central retinal artery. Blindness of this eye was immediate, absolute and permanent. A year afterward she comes to me with marked convergent strabismus. The blind eye has been seeking the position of repose in the direction of the least resistance. For cosmetic effect a wide and, so far as possible, complete division of the tendon of the internal rectus is made with satisfactory result. When repair of the wound is concluded a perceptible convergence may still be detected. No new forces have been at work to produce squint in this eye. Is it not reasonable to assume a decided unbalancing of the recti muscles, of long standing, but with proper coördination of movement as long as binocular vision existed? When one eye, however, is blinded, no incentive for properly directing this eye exists, and it gradually turns in as the compensating hypertrophy of the external rectus vanishes. How many graduated tenotomies, think you, would have sufficed to have established balance in this case had it been attempted for asthenopia?

Such meddlesome surgery as is exemplified in the first case mentioned, that of the asthenopic patient, does not, as a rule, work injury aside from loss of time, the dangers and inconvenience of operations and the depleting of the subject's pocketbook. Compensating development as surely remedies the errors of the tenotomist as it rights the wrongs of individual structure. But this can hold true of cautious surgery only, and, moreover, when directed solely to the external and internal recti muscles. If the obliques or superior or inferior recti be attacked disaster may follow. The following not unusual case illustrates such a calamity.

Mr. E. complains of asthenopia, and diplopia when looking below the horizontal plane. Examination, after employment of a mydriatic, reveals no important refractive error. He then gives this history: a year before, on leaving school where he had done little study, he entered a mercantile establishment and did much close work. His eyes became fatigued. For his asthenopia he consulted a gentleman of large practice and much experience in doing tenotomies. He was examined without a mydriatic; nothing was discovered in the way of a refractive error, though, of course, it might have existed in considerable degree. For latent hyperphoria he submitted to—first, tenotomy, sup. rect., right eye; second, tenotomy, sup. rect., left eye; third, tenotomy, inf. rect., left eye; fourth, tenotomy, inf. rect., left eye; fifth, galvanism locally, daily, for two weeks; sixth, tenotomy, inf. rect., left eye; seventh, advancement, inf. and sup. rect., left eye. If, standing, he looks at a candle placed on the floor six feet before him he sees two candles, one two feet above the other. The confusion is so intolerable that he is learning to keep one eye closed. I can suggest to him no remedy other than the production of a divergent squint, to put one eye entirely out of range and assist him in learning to suppress one image. Enucleation of one eye would simplify matters, but can hardly be considered; yet he is now liable to serious injury from falling on stairs and uneven surfaces. He had in the beginning a latent hyperphoria—that is, a tendency toward deviation of one eye upward, which he could and did correct. He now has a loss of the power to coördinate the downward movement of the visual axes. It is not now latent, for he cannot correct it, and it requires no phorometer or prisms to demonstrate it.

Now let me briefly state the method for treating these asthenopic patients which seems to me to be rational, and which, judging from results, I consider to be entirely adequate. First, however, in order that the importance of a proper treatment may be realized, I will say that fully three-quarters of my patients desire relief from asthenopia.

I first ascertain whether or not the general condition of the patient warrants the exertion he demands of his eyes. If it does I learn whether or not the ciliary muscle is correcting hypermetropia, hypermetropic astigmatism or presbyopia, in addition to its normal function of controlling accommodation. If deformity is excessive, *i. e.*, is more than is usual in the average man, glasses are ordered for close work or to be worn constantly, as the exigencies of the case demand. A mydriatic is necessarily used in all eyes not presbyopic. If the deformity be slight, the patient is told to get into training, assured that a physiological fatigue will limit overwork, but that bedridden eyes are frequently seen.

I have no desire to enter the lists to combat the intenable proposition that in graduated tenotomies of the ocular muscles we have a patent remedy for chorea, hystero-epilepsy, chronic constipation and insanity. All this and more has been asserted, but I believe it to be so absurd as to ensure its own destruction. However, such specious argument is made in so many quarters for tenotomies to relieve muscular asthenopia, so many patients are subjected to the risks of useless operations, and it all is, it seems to me, so far from truth, that I have, after observing in wonder and silence for many years, ventured to present the matter for your consideration.

With all due modesty, I wish to place myself on record in 1896 as saying that graduated tenotomy for the relief of asthenopia is, as a rule, irrational and dangerous.

53 SOUTH FITZHUGH STREET.

